# Methyl oleate deoxygenation for production of diesel fuel aliphatic hydrocarbons over Pd/SBA-15 catalysts

**DOI:** 10.1186/1752-153X-7-149

**Published:** 2013-09-05

**Authors:** Siew-Pei Lee, Anita Ramli

**Affiliations:** 1 Chemical Engineering Department, Universiti Teknologi PETRONAS, Bandar Seri Iskandar, 31750 Tronoh, Perak, Malaysia; 2 Fundamental and Applied Science Department, Universiti Teknologi PETRONAS, Bandar Seri Iskandar, 31750 Tronoh, Perak, Malaysia

## Abstract

**Background:**

Catalytic deoxygenation is a prominent process for production of renewable fuels from vegetable oil. In this work, deoxygenation of technical grade methyl oleate to diesel fuel aliphatic hydrocarbons (C_15_ – C_18_) is evaluated with several parameters including temperature, hydrogen pressure and reaction time in a stirred batch reactor over Pd/SBA-15 catalysts.

**Results:**

Two different SBA-15 morphologies i.e. spherelike and necklacelike structures have been synthesize as supports for Pd active metal. It is found that Pd dispersion on necklacelike SBA-15 is higher than that of spherelike SBA-15. Notably, higher Pd dispersion on necklacelike SBA-15 provides significant deoxygenation efficiency as compared to Pd/SBA-15-spherelike. Results show that H_2_ pressures greatly determine the total ester conversion and selectivity to C_15_ – C_18_ aliphatic hydrocarbons. Total ester conversions with 55< selectivity to *n*-heptadecane are achieved using Pd/SBA-15-necklacelike at 270°C and 60 bar H_2_ pressure within 6 h reaction time. Gas phase study reveals that formation of C_17_ is generated *via* indirect decarbonylation when the reaction time is prolonged.

**Conclusions:**

Pd/SBA-15-necklacelike catalyst exhibits good catalytic performance with high selectivity to diesellike aliphatic hydrocarbons (C_15_ – C_18_). The physicochemical properties of the Pd supported on different SBA-15 morphologies influence the deoxygenation activity of the catalysts. Furthermore, the reaction pathways are governed by the H_2_ pressure as well as reaction duration.

## Background

Currently, transportation fuel *i.e.* gasoline, diesel fuel and kerosene are obtained from crude oil refining [1]. Alternative fuels are being sought to overcome the problem associated with dwindling oil reserves and stringent environmental restrictions in greenhouse emissions. To date, government regulations strongly promote utilization of alternative fuels in the effort to reduce the green house gases (GHG) emission as underlined in Kyotol Protocol [2,3]. One of the approaches is to investigate the feasibility of vegetable oils to be the potential source for renewable fuels. Several technologies have been discovered to convert the triglycerides into biofuel like derivative of fatty acid methyl ester (FAME) from transesterification of fatty acid [4,5], hydrocracking of biofeedstock into fuel range hydrocarbons [6,7], hydrotreating of triglycerides and fatty acids into hydrocarbon middle distillates as well as production of renewable diesel followed by deoxygenation [8-11]. Although biodiesel production *via* transesterification is on commercial scale however, this process is highly sensitive to feedstock purity and the higher oxygen content of FAME exhibits poor fuel properties [12]. Thus, biodiesel produced is not compatible for the current combustion engine. Deoxgenation of biofeedstock *via* hydrodeoxygenation (HDO) or pyrolysis provides an alternative route to produce renewable fuels which can be used as a direct replacement for petroleum sourced hydrocarbons. Nowadays HDO process has been industrialized to form diesel fuel from vegetable oils. Removal of oxygen from triglycerides at elevated temperatures over heterogeneous catalyst in the presence of H_2_ produces long-chain alkanes from C_4_ to C_24_, typically C_16_ to C_18_[13-16].

Supported metal sulfides, NiMo and CuMo sulfides, are the industrialized catalysts for hydroprocessing of petroleum-based feeds. Similarly, these conventional hydrotreating catalysts have been utilized to up-grade vegetable oils to a hydrocarbon-based fuel [17]. Application of this process with the established existing hydroprocessing usually used in a petroleum refinery could eliminate the additional capital costs in construction of new infrastructure. However, metal sulfides used are required with careful handling for the process as well as has to mind for the risk of product with sulfur contamination [18].

Recently, development of sulfur free catalysts was the new approach for transformation of renewable sources into paraffinic liquid fuel. Senol *et. al.*[15] has reported that catalytic HDO with unsulfided NiMo catalyst is favoured in generating hydrocarbon s with odd number of carbon atoms while sulfided catalyst preferably yield hydrocarbons with even number atoms.

Murzin and co-workers have extensively investigated deoxygenation of triglycerides model compounds such as fatty acids and methyl esters over supported metal catalysts [11,19-21]. Commercial palladium (Pd) supported on activated carbon displayed as the most efficient catalyst in the deoxygenation of stearic acid at a reaction temperature of 300°C and 17 bar of 5 vol< H_2_/Ar^1^. Several reports revealed that supported Pd and Pt catalysts have high selectivity to form hydrocarbons with one carbon less than the corresponding fatty acids in the deoxygenation reaction while formation of CO and CO_2_ as the main gaseous products proposed that decarboxylation and decarbonylation were the primary reaction routes [21-25].

Immer *et al.*[26] provided insight for gas phase composition in catalytic deoxygenation of free fatty acids (FFA) using Pd/C catalysts. There was found that deoxygenation pathway is sensitive to the H_2_ partial pressure applied in the system. Madsen *et al.*[25] evaluated HDO activities for model fat mixture over Pt/γ-Al_2_O_3_ under different reaction conditions using a batch reactor and C_15_ – C_18_ diesel-like hydrocarbons were formed under H_2_ rich atmosphere.

To reduce the impact of internal mass transfer limitations and pore blockage, batch deoxygenation of ethyl stearate was studied using Pd supported on the siliceous mesocellular foam (MCF) [27]. However, the study showed that less than 15< conversion of ethyl stearate was observed at 300°C under N_2_ atmosphere.

Stucky and co-workers discovered hexagonal mesoporous molecular silica that is Santa Barbara amorphous No. 15 (SBA-15) in 1998 [28]. High surface area, large pore volume, high thermal and hydrothermal stabilities and well-defined pore size are the unique characteristics of SBA-15 to use as potential material in catalysis, adsorption, nanoelectronics and *etc*[29-31]. SBA-15 could be tailored out into different morphologies such as spheres [32], fibers [33] and rodlike [33] through monitoring of the synthesis conditions, thus having different physicochemical properties. The synthetic conditions including acid source [34,35], weight ratio P123/silica source [36], temperature [37,38], water content [39], pH value [39], addition of organic salt [38] and shearing flow [40] are the determining factors in fabrication of different forms of SBA-15. Differences in mesophase morphology may be significant for targeted application such as catalysis, separation, internal surface modification and bioimmobilization.

Pd/SBA-15 nanocomposite prepared by Wang *et al.*[41] demonstrated superior catalytic activity and recyclability for Heck carbon-carbon coupling reaction. Besides, Pd/SBA-15 was also reported showing superior catalytic activity in deoxygenation of stearic acid at 300°C under 17 bar of 5 vol< H_2_/Ar as compared to Pd/C, the conventional deoxygenation catalyst [42]. Hydrodeoxygenation of methyl oleate was studied over Ni_2_P/SBA-15 in a fixed-bed reactor and showed that uniform dispersion of NiP on SBA-15 resulted in higher conversion of esters and selectivity of long-chain paraffin [16].

Supported Pd on SBA-15 with 3 wt< as well as 5 wt< were tested for deoxygenation of stearic acid. The catalyst with 3 wt< showed highest catalytic activity and provided 90<*n*-heptadecane selectivity [42]. Nevertheless, it found that 3.8 wt< supported Pd on SBA-15 was the optimum Pd loading for deoxygenation of methyl oleate [43]. In this regard, catalytic deoxygenation of methyl oleate to C_15_ – C_18_ aliphatic hydrocarbons which are the major components find in diesel fuel is investigated over supported 3.8 wt< Pd nanoparticles on two different SBA-15 mesostructures, necklacelike and spherelike, using batch stirred reactor. Methyl oleate is selected as a feedstock for this study is due to presence of major amount in fatty acid monoesters molecules which are mostly found in vegetable oil.

## Results

### Phase structure and physicochemical characterization

**Figure 1:**
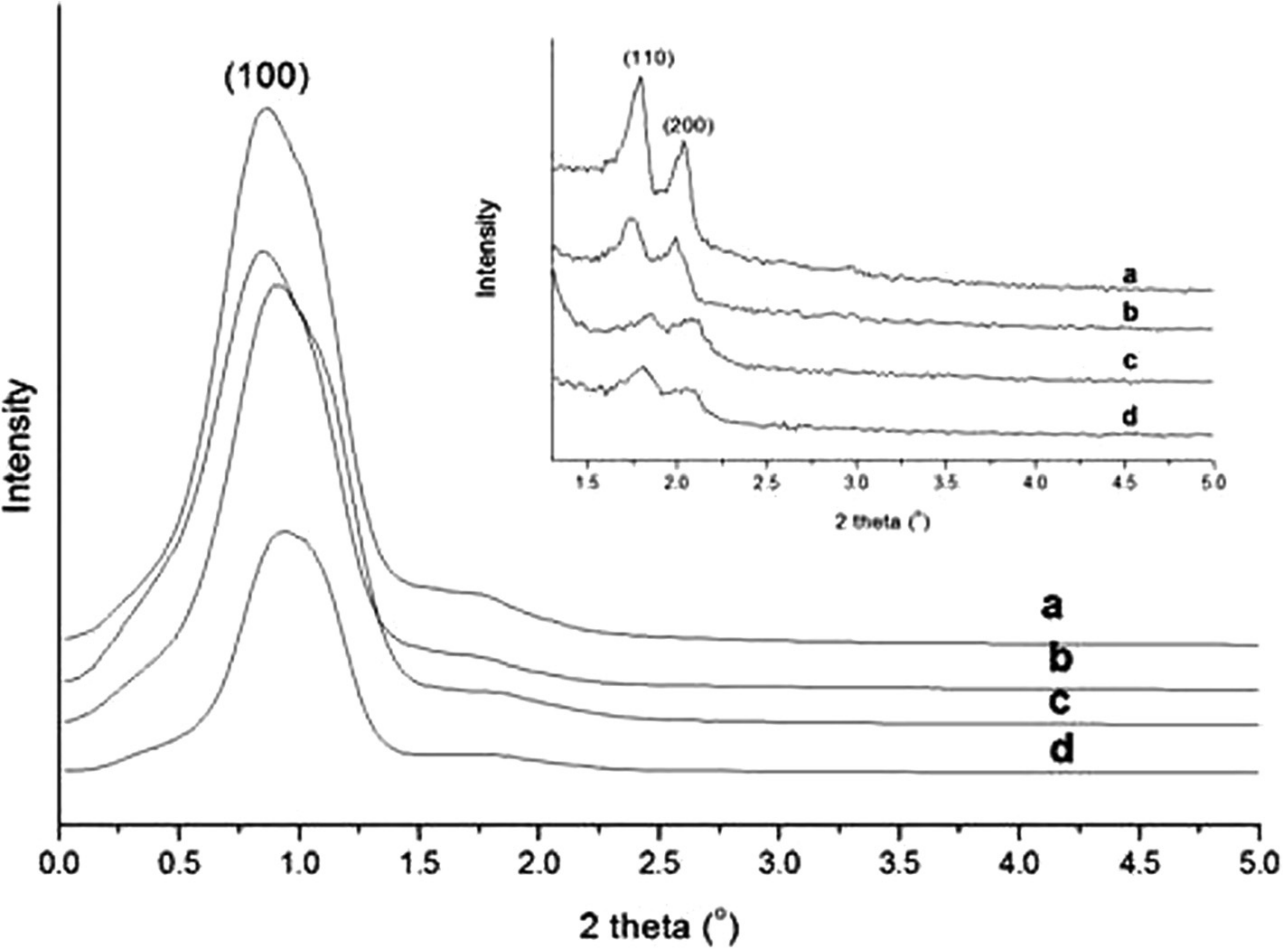
**XRD patterns at low diffraction angles for Pd supported on SBA-15 structure. (a)** SBA-15-necklacelike calc., **(b)** 3.8 wt< Pd/SBA-15-necklacelike **(c)** SBA-15-spherelike calc., **(d)** 3.8 wt< Pd/SBA-15-spherelike. Insert shows the local magnification of small-angle X-ray diffraction patterns for Pd/SBA-15.

Small angle X-ray Diffraction (XRD) patterns of SBA-15 and Pd/SBA-15 samples synthesized are showed in Figure 1. An intense peak in the XRD pattern at 2Θ within 0.8 – 0.9^o^ (Figure 1) and two well-resolved peaks at 1.6 – 2.0^o^ (Figure 1 insertion) are the (100), (110) and (200) diffractions of the ordered hexagonal lattice (*p6mm*) with continuous silica walls of SBA-15.

**Figure 2:**
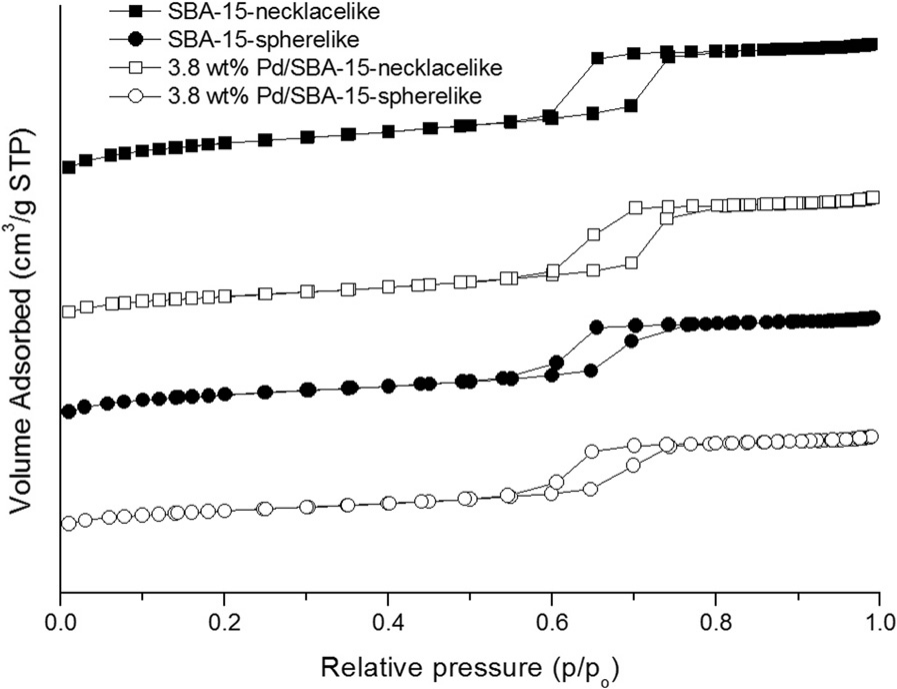
**N**_**2 **_**adsorption desorption isotherms of SBA-15-calc. and Pd/SBA-15-calc.** They are offset vertically by 300 cm^3^ g^-1^ STP^-1^ for clarity.

Figure 2 shows the nitrogen adsorption-desorption isotherms for siliceous SBA-15 and Pd/SBA-15 samples. Both necklacelike SBA-15 and spherelike SBA-15 samples exhibits type-IV isotherms according to International Union of Pure and Applied Chemistry (IUPAC) classification with type-H1 hysteresis loop which is a typical characteristics exhibited by mesoporous silica.

The textural properties such as Brunauer-Emmet-Teller (BET) surface area, average pore diameter and cumulative adsorption pore volume obtained by Barret-Joyner-Halenda (BJH) are given in Table 1. SBA-15 materials prepared show high specific surface area in the range of 783 – 432 m^2^/g and high specific pore volume with 0.88 – 0.67 cm^3^/g.

**Table 1:** **The main characteristics of mesoporous silica SBA-15 and Pd/SBA-15 composites: S**_**BET**_**, specific surface area (m**^**2**^**/g), V**_**p**_**, pore volume (cm**^**3**^**/g), D**_**p**_**, average pore diameter (nm)**

**Sample**^**a**^	**Actual Pd content**^**b **^**(wt<)**	**S**^**c**^_**BET **_**(m**^**2**^**/g)**	**V**_**p**_^**d **^**(cm**^**3**^**/g)**	**D**_**p**_^**d **^**(nm)**	**Pd dispersion**^**e **^**(<)**
SBA-15-N	-	783	0.88	5.6	-
3.8 wt< Pd/SBA-15-N	3.2	482	0.81	6.4	39.0
SBA-15-S	-	561	0.68	5.6	-
3.8 wt< Pd/SBA-15-S	3.7	432	0.67	6.0	19.0

SBA-15-N: necklacelike SBA-15; SBA-15-S: spherelike SBA-15.

^a^Nominal palladium loading.

^b^Determined by ICP-MS.

^c^Calculated by BET method.

^d^Calculated from adsorption branch by BJH method.

^e^Measured by H_2_ chemisorption.

BET surface area for both SBA-15 morphologies decreases significantly with Pd incorporation although minor changes in the specific pore volume are observed.

**Figure 3:**
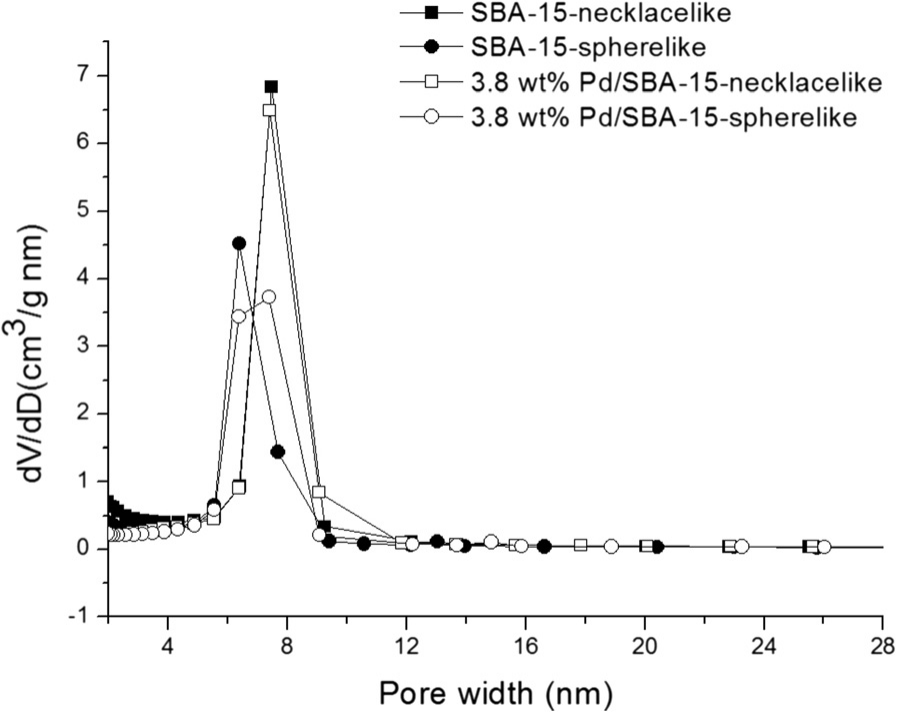
Pore size distribution of SBA-15-calc. and 3.8 wt< Pd/SBA-15-calc.

The pore size distribution curves of short spherelike and necklacelike samples which are calculated from adsorption branch by BJH shows narrow pore size distribution around a diameter of 5.5 – 6.5 nm (as shown in Figure 3). However wider distribution in the pores of Pd/SBA-15 samples is observed. As a result, the average pore diameter reported is bigger than that of parent SBA-15 (Table 1).

**Figure 4:**
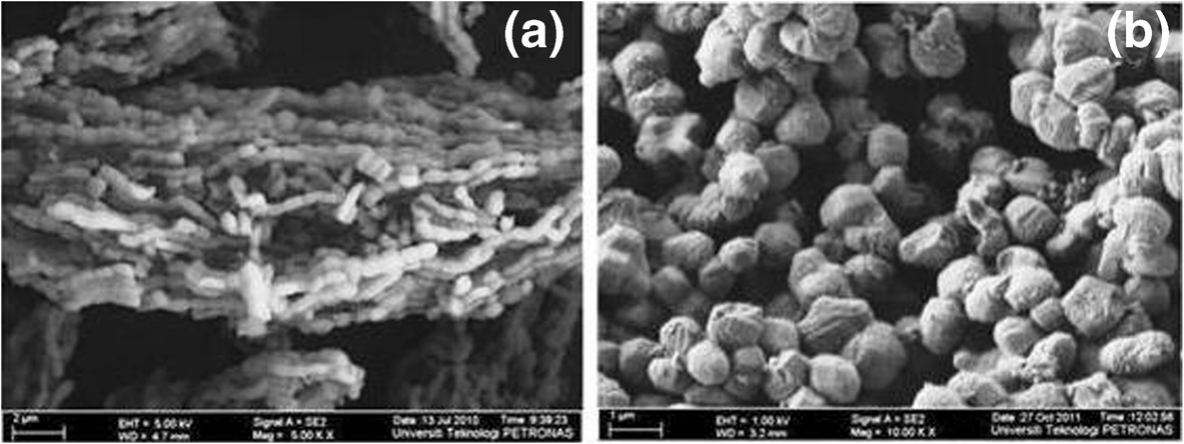
**FESEM images of the SBA-15 catalysts. (a)** necklacelike SBA-15 and **(b)** spherelike SBA-15.

**Figure 5:**
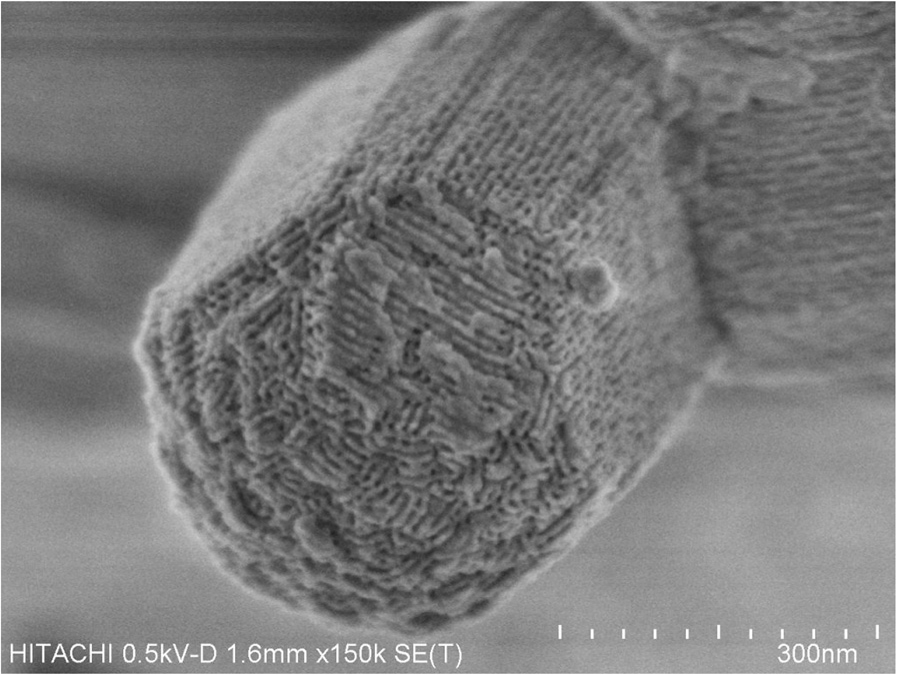
Low accelerating FESEEM image of SBA-15 with honeycomb disposition of pore entrance.

Field Emission Scanning Electron Miscroscopy (FESEM) image shows the formation of different lengths of necklacelike SBA-15 which possess bundles of micro-sized rod (Figure 4a). On contrary, individual spherelike particles are observed from Figure 4b. High resolution FESEM micrograph in Figure 5 clearly shows that necklacelike SBA-15 mesoporous silica is packed with well-ordered 2D hexagonal array.

**Figure 6:**
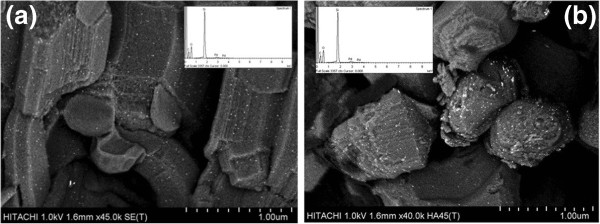
**Low accelerating FESEM voltage images of Pd/SBA-15. (a)** Pd/SBA-15-necklacelike and **(b)** Pd/SBA-15- spherelike. Insert shows the EDX patterns of Pd/SBA-15.

**Figure 7:**
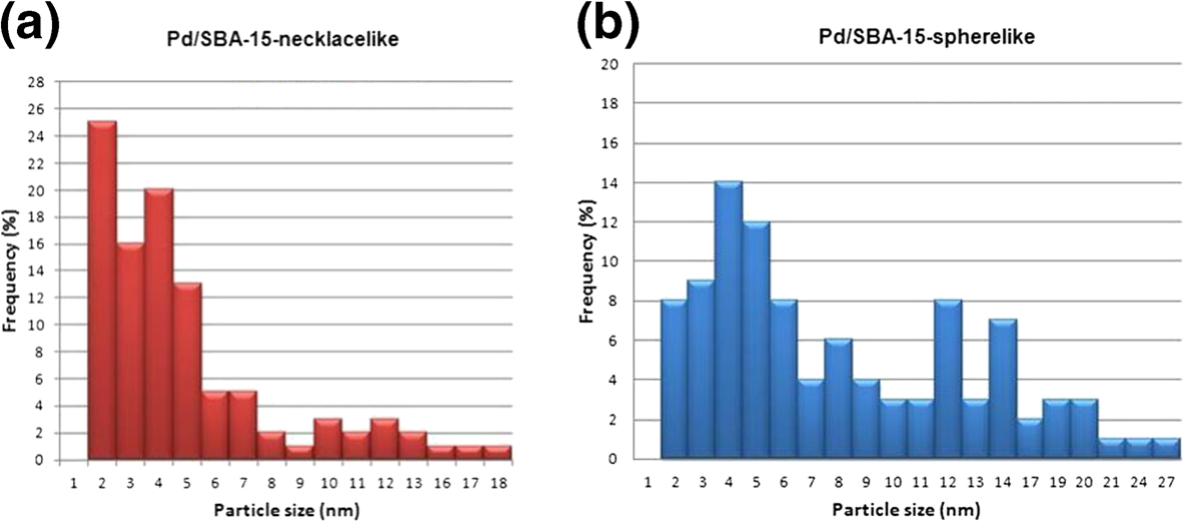
**Particles distribution of Pd/SBA-15. (a)** Pd/SBA-15-necklacelike and **(b)** Pd/SBA-15- spherelike.

Low acceleration voltage FESEM and Energy-dispersive X-ray (EDX) pattern of Pd/SBA-15 are presented in Figure 6 (a) and (b). It is observed that PdO nanoparticles are distributed on external surface of SBA-15 where the presence of Pd element is further confirmed from the Pd signals generated in EDX pattern [inserted Figure 6 (a) & (b)]. Figure 7 (a) and (b) shows the particle size distribution derived from the low acceleration voltage FESEM images by surveying 100 particles. It should be noted that the sizes of the PdO nanoparticles formed on the external surface of necklacelike SBA-15 are distributed uniformly in a range of 2 – 5 nm [Figure 7 (a)]. Thus, Pd/SBA-15-necklacelike has a narrow particle size distribution with largest size of 18 nm. In comparison, a broader particle size distribution is observed for Pd/SBA-15-spherelike and the largest particle size measured is 27 nm.

H_2_ pulse chemisorption is used to measure Pd dispersion adsorbed on SBA-15 surface (Table 1). 39< Pd dispersion is found on necklacelike SBA-15 however, spherelike SBA-15 only provides 19< Pd dispersion.

### Catalyst evaluation

Liquid phase deoxygenation of triglyceride surrogate molecules, methyl oleate to C_15_ – C_18_ aliphatic hydrocarbons is investigated in a stirred batch reactor over 3.8 wt< Pd/SBA-15 on two different SBA-15 morphologies as discussed before. Catalytic deoxygenation of methyl oleate (70< technical grade) is studied using Pd/SBA-15-necklacelike (N1) and Pd/SBA-15-spherelike (S1) catalysts at 270°C, 60 bar H_2_ pressure, catalyst loading of 3 wt< and 6 h reaction duration.

**Figure 8:**
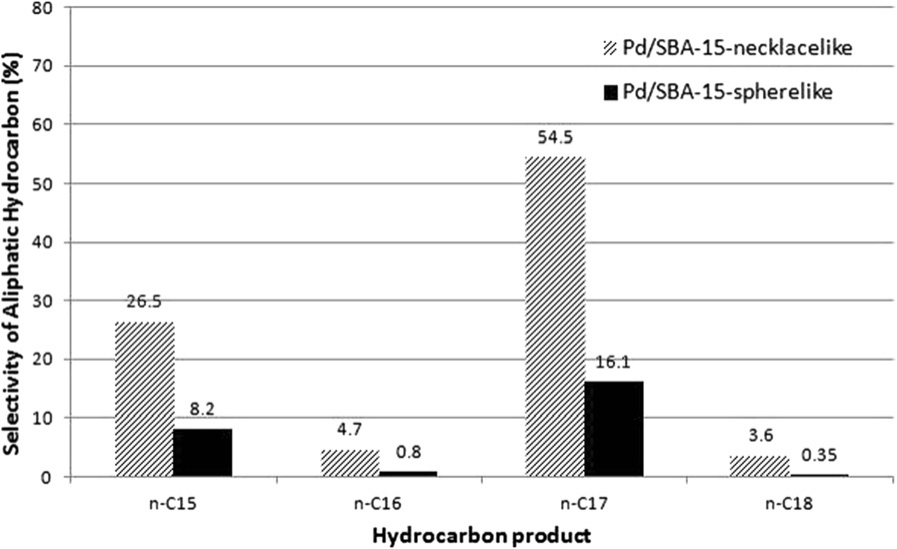
**Comparison of the selectivity for C**_**15 **_**– C**_**18 **_**aliphatic hydrocarbon over Pd/SBA-15-necklacelike and Pd/SBA-15-spherelike catalysts at 60 bar H**_**2 **_**pressure and 270°C within 6 h reaction time.**

The feedstock used for the experiments is a technical grade of methyl oleate, containing a mixture of 76<, methyl oleate, 5< of methyl palmitoleate and 18< of methyl palmitate. Similar total ester conversion that is about 95< as shown in the Table 2 is observed for both catalysts since majority of unsaturated esters are converted at 60 bar H_2_ pressure. Formation of saturated esters was reported to be more prominent under high H_2_ pressure [21]. However, higher Pd dispersion on necklacelike SBA-15 that is 39< provides superior selectivities for C_15_ to C_18_ aliphatic hydrocarbons as compared with Pd supported on spherelike structures (Figure 8). Result shows that 55< selectivity to *n*-heptadecane was achieved using Pd/SBA-15-necklacelike catalyst as compared to only 16< selectivity shown by Pd/SBA-15-spherelike catalyst, indicating that Pd dispersion has significant influence in the catalyst performance.

**Table 2:** Total ester conversion with supported Pd on two different morphologies of SBA-15

**Catalyst**	**BET surface area (m**^**2**^**/g)**	**Pd dispersion**^**a **^**(<)**	**Total ester conversion (<)**
N1	482.2	39.2	95
S1	431.9	18.5	94.3

^a^Metal dispersion determined by H_2_ pulse chemisorptions.

N1: 3.8 wt< Pd/SBA-15-necklacelike.

S1: 3.8 wt< Pd/SBA-15-spherelike.

**Figure 9:**
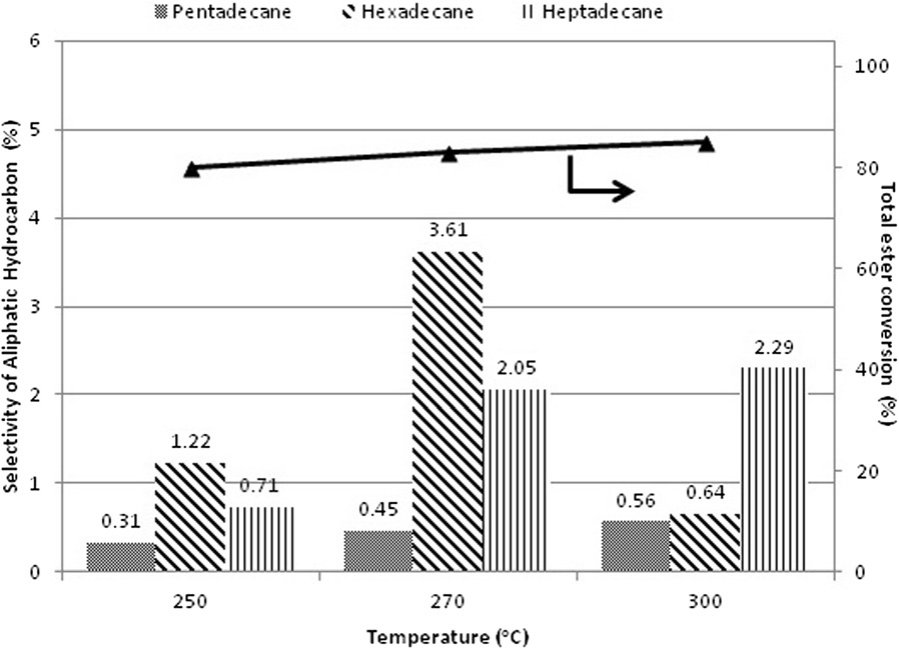
**Selectivity for C**_**15 **_**– C**_**17 **_**aliphatic hydrocarbon and total ester conversion with reaction temperature variation over Pd/SBA-15-necklacelike at H**_**2 **_**pressure 25 bar in 3 h reaction time.**

The effect of temperature on the catalytic activity is investigated using Pd supported by necklacelike SBA-15. Figure 9 shows the selectivity to C_15_ to C_17_ aliphatic hydrocarbons is low within 3 h duration over the temperature range from 250 - 300°C at 25 bar H_2_ pressure. However, formation of *n*-octadecane was not observed in the product mixture within 3 h of reaction.

**Figure 10:**
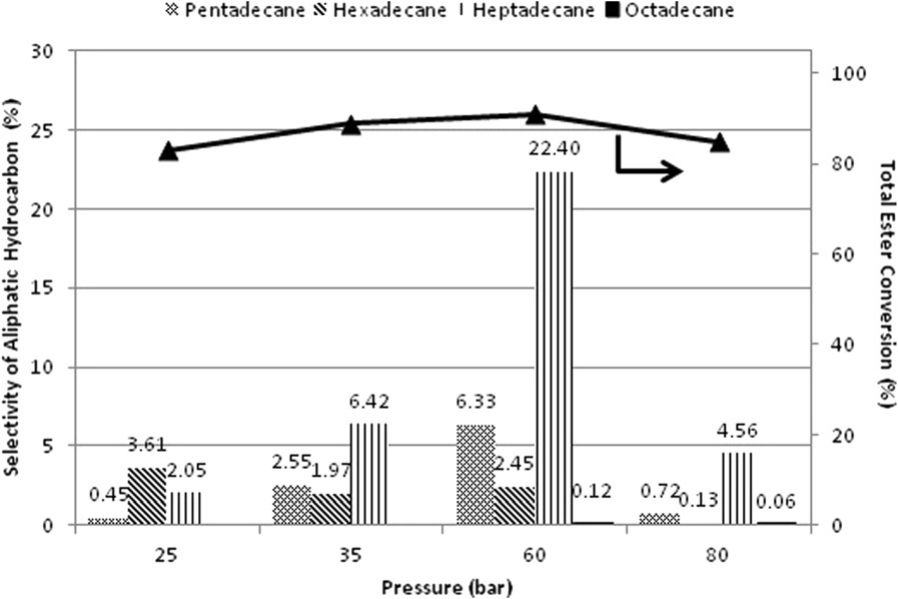
**Selectivity for C**_**15 **_**– C**_**18 **_**aliphatic hydrocarbon and total ester conversion with variation of H**_**2 **_**pressure over Pd/SBA-15-necklacelike at T = 270°C within 3 h reaction time.**

Effect of hydrogen pressure on the selectivity of linear hydrocarbon is studied (Figure 10). Reaction temperature of 270°C is selected for further investigations due to the total yield of paraffinic hydrocarbons obtained is higher in amongst of temperature investigated. It is important to notice that ester conversion and selectivity of paraffin are sensitive to pressure variation at 270°C (Figure 10). Accordingly, increment of the total ester conversion and selectivity of C_15_ to C_18_ paraffinic hydrocarbons are in parallel with the reaction pressure from 25 bar to 60 bar H_2_ pressure. The selectivity for C_15_ to C_18_ long chain hydrocarbons is relatively low from 25 bar to 35 bar H_2_ pressure as compared with 60 bar H_2_ pressure at 270°C within 3 h reaction duration. At 60 bar, the selectivity for *n*-heptadecane is exceptionally high than other aliphatic hydrocarbons at 60 bar H_2_ pressure. On the other hand, a decrease in ester conversion from 60 to 80 bar H_2_ pressure and suppression for yield of *n*-alkanes formation are observed. Obviously, deoxygenation of methyl oleate using Pd/SBA-15 is less pronounced at 80 bar.

**Figure 11:**
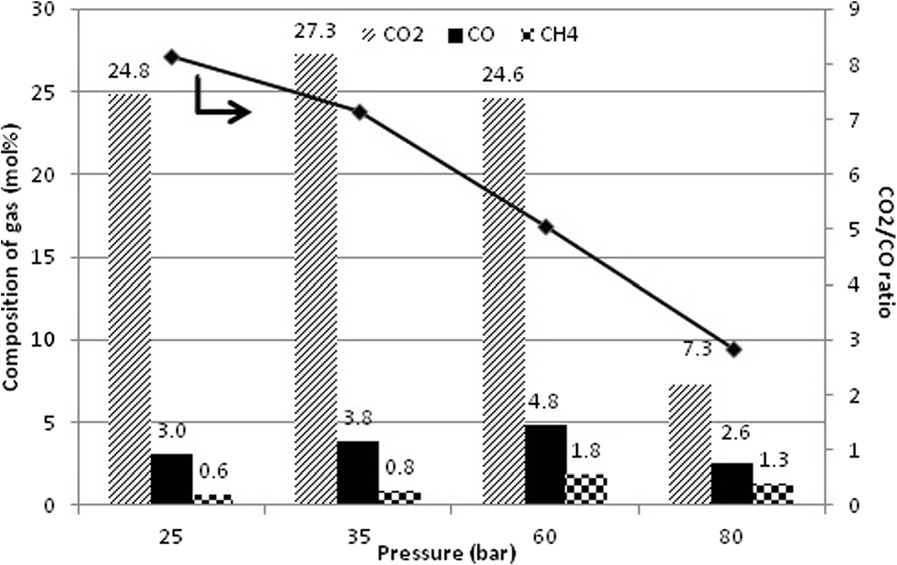
**Composition of main carbonaceous gases detected and CO**_**2**_**/CO ratio with over 3.8 wt< Pd/SBA-15-necklacelike at 60 bar H**_**2 **_**pressure and T = 270°C.**

Figure 11 shows that the composition of carbonaceous gases for different pressure at 270°C in H_2_. Still, CO_2_ is detected as the major carbonaceous gas product. However, CO_2_/CO ratio decreases when H_2_ pressure increases (Figure 11). The concurrent reduction of the CO_2_ concentration and increment of methane formation as increasing of H_2_ pressure may be due to the hydrogenation of CO_2_ to methane.

**Figure 12:**
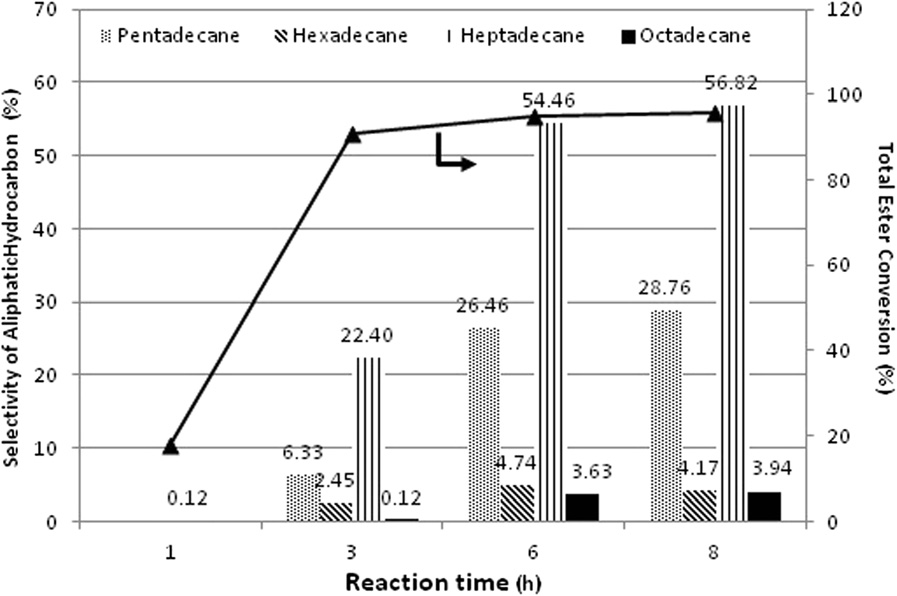
**Selectivity for C**_**15 **_**– C**_**18 **_**aliphatic hydrocarbon and total ester conversion with time variation over Pd/SBA-15-necklacelike at 60 bar H**_**2 **_**pressure and 270°C.**

Batch deoxygenation of mehyl oleate with reaction duration is further attempted at 270°C and 60 bar H_2_ using 3.8 wt< Pd/SBA-15 necklacelike catalyst (Figure 12). The catalyst is exceptionally active for hydrocarbons production when the reaction duration are extended to 8 h since the selectivity to C_15_ – C_18_ hydrocarbons increases up to approximately 57< (Figure 12). Total esters conversion attained 95< but it is reached a plateau after 6 h at 270°C and 60 bar H_2_ pressure. But no significant selectivity improvement for C_15_ to C_18_ long chain hydrocarbons is observed after 8 h of reaction.

**Figure 13:**
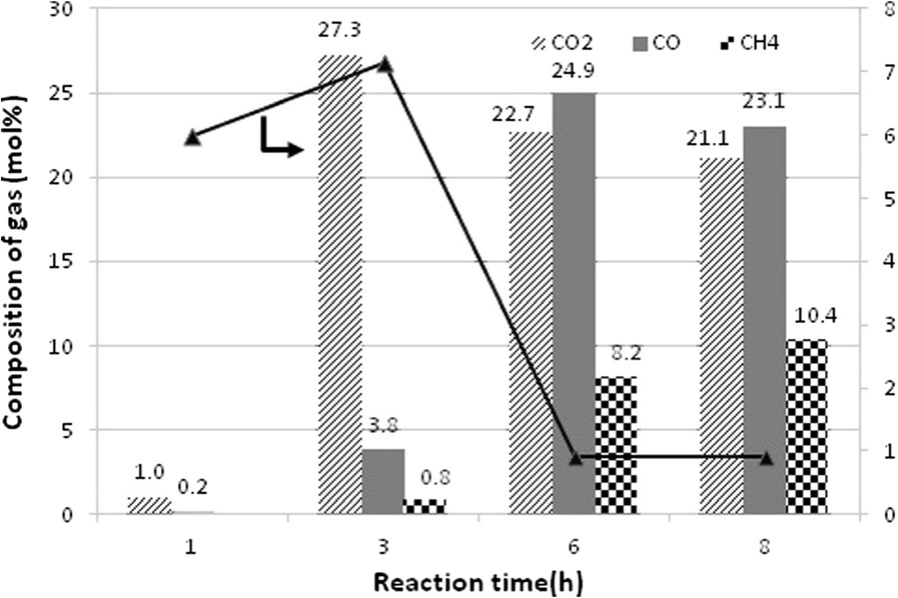
**Composition of main carbonaceous gases detected and CO**_**2**_**/CO ratio with over 3.8 wt< Pd/SBA-15-necklacelike at 60 bar H**_**2 **_**pressure and T = 270°C.**

At 270°C and 60 bar hydrogen pressure, the selectivity of *n*-heptadecane was relatively low during the reaction time of 1 h and no others paraffinic hydrocarbons are found in the reaction mixture (Figure 13). Solely *n*-heptadecane is found during 1 h reaction time may imply that decarboxylation or decarbonylation is predominant in the initial reaction stage. It should, however, be noted that, no aromatic and unsaturated C_17_ compounds are detected in all time stream.

Figure 13 shows the evolution of gas-phase products with reaction time on 3.8< Pd/SBA-15-necklacelike at 270°C and 60 bar H_2_ pressure. CO_2_, CO and CH_4_ are evolved concomitantly during the reaction progression. Unlike the increase in the concentration of CO with time, the CO_2_ evolution decreases monotonically with reaction time. CO_2_/CO ratio is increased within 3 h reaction while subsequently there is a sharp decrease in CO/CO_2_ ratio (Figure 13). Besides, the methane content is increased remarkable in the total of carbonaceous gas products from 6 to 8 h reaction (Figure 13).

## Discussion

Ordered hexagonal pore array structure of SBA-15 with Pd incorporated is preserved since no significant change between the diffraction patterns which indexed as (100), (110) and (200) for Pd/SBA-15 and SBA-15 materials (Figure 1). However, it is observed that the intensity of the diffraction peaks at 2θ between 1.6 – 2.0° decreases significantly in necklacelike structure as compared to spherelike structure (inserted Figure 1). A decrease in peak intensity is attributed of distribution of the Pd on the external surface of the SBA-15. This means that more Pd particles are distributed on the external surface of necklacelike structure as compared to spherelike structure, thus resulted in a more significant reduction in surface area of necklacelike structure compared to spherelike structure.

A shift of the diffraction peaks to lower angles is identified from Pd/SBA-15 samples, reflecting an increase in unit cell parameter of the SBA-15 framework. As reported by other researchers [44-46], this feature is because of the metals substitution into mesoporous molecular sieves. Therefore, substitution of Pd into SBA-15 framework might be the main reason to increase the unit cell parameters of the SBA-15 mesoporous structure.

Hysteresis loop as observed in Figure 2 is due to the N_2_ capillary condensation in cylindrical mesoporous channels. Besides, no significant change of isotherm pattern from Pd/SBA-15 samples suggests that SBA-15 template is preserved with anchoring of Pd ion *via* impregnation. Necklacelike SBA-15 synthesized provides larger surface area and higher pore volume than spherelike SBA-15. This may be attributed to the formation of larger micelles quantity as a result of stirring during synthesis of necklacelike SBA-15. The micelles are then removed during calcination resulting in an increase in the number of pores thus an increase in the total surface area and total pore volume [32].

The significant reduction in surface area without significant decrease in pore volume (Table 1) may indicate that most of the PdO particles are distributed on the external surface of the support as compared to in the pores, thus resulted in a more significant reduction in the surface area as compared to the reduction in pore volume. However, wider pore distribution for Pd/SBA-15 samples was observed after incorporation of Pd in SBA-15 (Figure 3). As a result, the slight increase in average pore size of Pd-incorporated catalyst is due to an increase in pore distribution.

Better Pd distribution on necklacelike SBA-15 as compared to spherelike SBA-15 (Table 1) due to the most of the smaller PdO particles, ranged in 2–5 nm, are being formed on the surface of the necklacelike structure [Figure 7 (a)]. This may be attributed the larger surface area available in the necklacelike support, thus enable a better Pd dispersion. In addition, larger particle size found on spherelike SBA-15 is resulted from the agglomeration of PdO nanoparticles during calcinations process.

Stirring flow applied in reaction mixture governs the formation of different SBA-15 morphologies [32,39]. Exerted shearing stress from continuous stirring promotes intermicelle interactions while short cylindrical silicate-surfactant micelles are lengthened along the flow direction and joined into longer silicas structure [Figure 4 (a)]. In addition, higher intensity of diffraction peaks at 2θ between 1.6 – 2.0° is observed for necklacelike SBA-15 as compared to spherelike SBA-15 (inserted Figure 1). Notably, the crystallinity of necklacelike SBA-15 is increased as the synthesis temperature was raised to 80°C. Therefore, it found that elongation of the longer silicas rod units under higher temperature leads to the formation of necklacelike SBA-15 structure.

It is found that better Pd/SBA-15-necklacelike contributes higher deoxygenation efficiency for aliphatic hydrocarbon formation instead of Pd/SBA-15-spherelike (Figure 8). The increased of surface-to-volume ratio due to higher Pd dispersion on necklacelike SBA-15 may enhance the adsorption of active Pd metal to methyl oleate molecules.

Pd/SBA-15-necklacelike catalyst is considered active over the temperature range studied as the total esters conversion achieved is more than 80< after 3 h reaction (Figure 9). It is observed that majority of methyl octadecanoate and methyl hexadecanoate are formed *via* hydrogenation of the unsaturated methyl esters as the reaction intermediates. Besides, the off-line gas analysis shows that CO_2_ is the main carbonaceous gas product.

**Figure 14:**
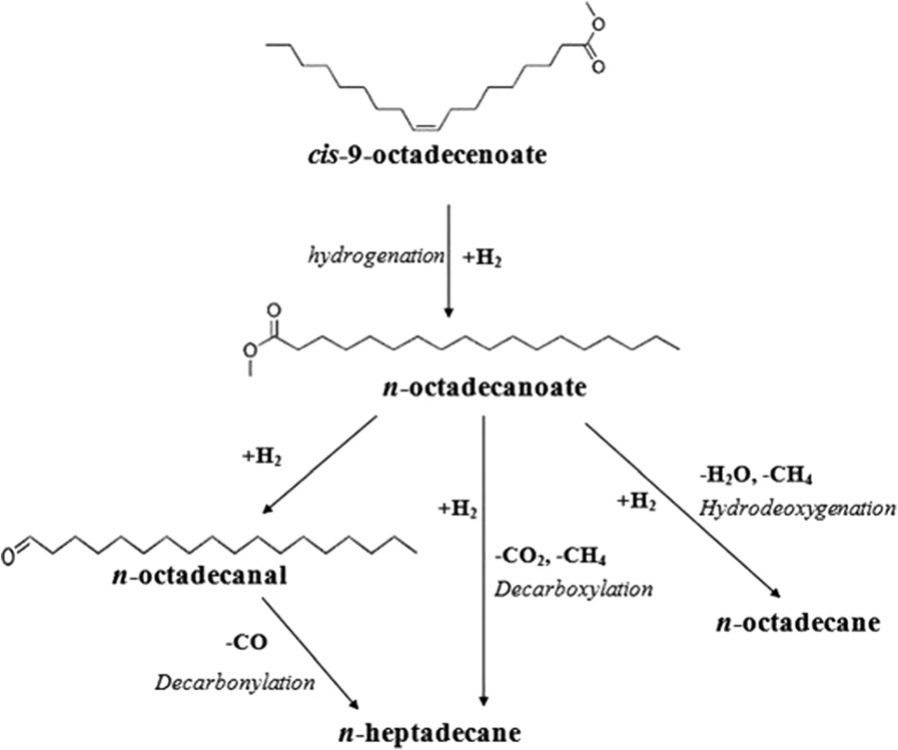
Catalytic pathways of methyl oleate deoxygenation over Pd/SBA-15 catalyst.

Hydrogenation of double bond in methyl esters molecules proceed faster than deoxygenation of the methyl esters functionalities within 250 – 300°C in 3 h reaction period. Majority formation of CO_2_ in a gas phase reveals that supported Pd catalyst is active and selective to produce *n*-heptadecane *via* decarboxylation instead of hydrodeoxgyenation (as shown in Figure 14).

Low selectivity of C_15_ to C_18_ aliphatic hydrocarbons (Figure 10) is due to saturation of dissolved H_2_ in reaction medium under high H_2_ pressure (80 bar), which active metal Pd interaction with methyl oleate molecules is inhibited like described by Madsen *et al.*[25]. Reaction moves toward decarbonylation route in a presence of higher H_2_ pressure results decrease of CO_2_/CO ratio (Figure 11). The switchover phenomenon observed enhances the finding reported by Boda *et al*. [8] and Immer *et al.* studies [26]. It is expected that methanation is facile under high H_2_ pressure condition like other reports mentioned [47]. But it might be possible that minor methane formation is come from the CO hydrogenation as suggested by Donnis *et al.*[10].

55< selectivity to *n*-heptadecane in 6 h reaction time demonstrates that Pd supported on necklacelike SBA-15 exhibits high deoxygenation efficiency to methyl oleate (Figure 12). In comparison to the work reported by Snare *et al.*[21], only about 25< selectivity to the main product *n*-heptadecane was obtained from technical grade of methyl oleate using microporous Pd/C. Therefore, it is true to some extent that Pd/SBA-15 catalyst outperforms than Pd/C either using complex mixture of unsaturated esters in the present study or pure stearic acid itself [42]. However, insufficient contact time for interaction of active metal Pd and methyl esters molecules into paraffinic hydrocarbons gives low selectivity of *n*-heptadecane in 1 h reaction.

As indicated in Figure 12, Pd supported on SBA-15 does not promote deoxygenation of fatty acids or ester functionalities *via* HDO pathway. In contrast, supported Pd catalyst is active in decarboxylation and/or decarbonylation route for elimination of C = O molecules to form odd-carbon-number of hydrocarbons. Isomers and unsaturated hydrocarbons are absence since Pd has been known as the strong hydrogenation agent. It is notably that isomerization and dehydrogenation are hardly involved in H_2_ atmosphere [11].

Production of CO and CO_2_ during decarboxylation/decarbonylation is crucial to understand plausible reaction path for the production of linear hydrocarbons. *n*-alkanes formation are preferable *via* decarboxylation within 3 h initial reaction time (as shown in Figure 13). However, highly dissolved H_2_ in the alkane solvent in a longer reaction time under high H_2_ pressure may push either decarbonylation or reverse water gas shift equilibrium towards increased CO formation in a gas phase. As previously showed in Figure 12 selectivity of *n*-heptadecane is significantly increased when reaction time is prolonged. As a result, switchover product selectivity from CO_2_ to CO observed reveals that conversion of methyl oleate to *n*-heptadecane is predominant *via* indirect decarbonylation. Methyl stearate, hydrogenated methyl oleate, proceeds *via* partial reduction of the carboxylic group to corresponding *n*-octadecanal followed by released of CO to form *n*-heptadecane as increasing of time-on-stream (Figure 14). High methanation of CO_2_ or CO attributes significant amount of methane in a gas phase as increased of reaction time. This finding shows that Pd/SBA-15 is active to reduce fully the oxidized carbon into methane.

## Conclusions

Necklacelike SBA-15 with high surface area is a great support to disperse Pd nanoparticles. Nanosized Pd particles anchored on necklacelike SBA-15 shows better deoxygenation reactivity than Pd/SBA-15-spherelike catalyst. Exceptionally high conversion of 70< technical grade of methyl oleate into diesel fuel aliphatic hydrocarbons (C_15_ – C_18_) is achieved at mild temperature *i.e.* 270°C and high H_2_ pressure, 60 bar in 6 h reaction time using batch reactor. Indirect decarbonylation pathway is dominant as increasing of reaction time. Here is found that Pd/SBA-15 is keenly involved in methanation during reaction progression. Deoxygenation of methyl oleate, surrogate molecule of vegetable oil, in the present work is greatly influenced by different support morphologies and reaction parameters.

## Methods

### Catalyst preparation

Necklacelike mesoporous SBA-15 was prepared by dissolving Pluronic P123 (PEO20PPO70PEO20) (Aldrich) in 2 M hydrochloric acid (HCl) (Merck) at 35°C before addition of tetraethylorthosilicate (TEOS) (Merck). The reaction mixture was then continuously stirred at 35°C for 24 h. Next, the solution temperature was raised to 80°C and stirred another 48 h. Meanwhile, spherelike SBA-15 was prepared by vigorously stirring the reaction mixture at 35°C for 10 minutes. Subsequently, the solution mixture was kept in isothermally static condition for 6 h. Template removal of SBA-15 was achieved by calcination in air at 550°C for 4 h. SBA-15 supported palladium catalyst, Pd/SBA-15 with 3.8 wt< Pd was prepared by impregnation method, filtered, air dried and calcination in air at 550°C for 4 h.

### Catalyst characterization

The concentration of Pd (wt<) incorporated into SBA-15 was measured by Inductively Coupled Plasma-Mass Spectrometer (ICP-MS), Agilent 7500 series. The BET surface area and pore diameters of the samples were determined by nitrogen adsorption-desorption isotherms measurement at 77 K using Quantachrome Autosorb 6B. XRD patterns of the catalysts were recorded with Bruker AXS D8 Discover equipped with general area diffraction detector system (GADDS) working with CoKα1 (1.5418 Å). In order to obtain higher solution of low angle XRD patterns, all the calcined samples were recorded on Bruker D8 Advance Powder diffractormeter (CuKα1 radiation, λ = 1.5406 Å).

The metal dispersion of supported palladium catalysts were investigated by H_2_ chemisorption. Pulse chemisorption on reduced palladium catalysts was performed at 70°C using Temperature Programmed Desorption Reduction and Oxidation (TPDRO) 1100 (Thermo Finnigan). The number of exposed palladium atoms on the surface was calculated by the total amount of hydrogen adsorped using the molar ratio between Pd:H_2_ as 0.5 [19].

FESEM coupled with EDX analysis was performed on Supra 55 VP, Carl Zeiss scanning electron microscopy. Low accelerating voltage FESEM image of the catalysts were taken using Hitachi SU-8000.

Transmission Electron Microscope (TEM) micrographs were captured by Carl Zeiss LIBRA 200 (with an accelerating voltage 200 kV).

### Catalyst evaluation

Evaluation of the Pd/SBA-15 performance in deoxygenation reaction was carried out in a 200 ml Hastelloy autoclave. The catalysts were primarily reduced with 5 vol< H_2_/Ar at 300°C for 1 h while, the autoclave was charged with 10 wt< of model feedstock, methyl oleate (70< Aldrich) in *n*-heptane. Then, the active catalysts were charged in the reactor vessel under inert atmosphere and the reaction was conducted at temperature between 250 – 300°C and H_2_ pressure range from 25 to 80 bar. Liquid reaction samples were analyzed offline using gas chromatography coupled with mass spectrometer (Shimadzu QP5050) using HP-5 column. Gas samples were taken out from the autoclave in a 100 ml gas tank and injected with a gas syringe on a gas chromatography (Agilent, 7890A) equipped with a thermal conductivity detector (TCD), split/splitless injection system and Porapak Q column.

## Competing interests

Both authors declare no competing financial interest.

## Authors’ contributions

This research conception is carried out in constructive discussions between SPL and AR. SPL who carried out the synthesis, characterization and deoxygenation studies wrote out the content of manuscript based on the analysis and data interpretation. AR was involved in revising this manuscript critically for key intellectual content. Both authors read and approved this final manuscript before submission.
